# Intestinal Hemangiomatosis: Case Report of an Uncommon Cause of Rectal Bleeding

**DOI:** 10.5334/jbsr.3072

**Published:** 2023-02-22

**Authors:** Rita Pina-Prata, Carina A. Ruano, Vera B. Carvalho, Ana Nunes, Eugénia Soares

**Affiliations:** 1 Radiology Department, Centro Hospitalar Universitário de Lisboa Central – Hospital Dona Estefânia, Lisbon, Portugal; 2 Radiology Department, Centro Hospitalar Universitário de Lisboa Central – Hospital de Santa Marta, Lisbon, Portugal; 3 Hospital Dona Estefânia, CHULC, Lisbon, Portugal

**Keywords:** Pediatrics, Radiology, Gastroenterology, Hemangioma, Intestinal

## Abstract

A four-month-old girl presented with recurrent low gastrointestinal hemorrhage. Abdominal ultrasound showed diffuse parietal thickening and hyperemia of the colon. Computed tomography (CT) demonstrated diffuse thickening of the colon but also intense arterial globular mural enhancement with diffuse filling in the portal phase. Colonoscopy revealed multiple pseudopolipoid lesions along the colon which were histologically diagnosed as hemangiomas. The infant was diagnosed with gastrointestinal hemangiomatosis and was treated with propranolol resulting in complete resolution of symptoms.

**Teaching point:**

Although rare, the possibility of intestinal hemangiomatosis should be considered in the setting of rectal bleeding in an infant.

## Introduction

Intestinal hemangiomatosis affects primarily infants or children and consists of multiple or large intestinal hemangiomas. The most common clinical presentation is gastrointestinal bleeding [1,2].

## Case History

A four-month-old girl was referred to our institution for recurrent low gastrointestinal hemorrhage (first episode at the seventh day of life). Physical examination and laboratory data were unremarkable.

**Figure 1:**
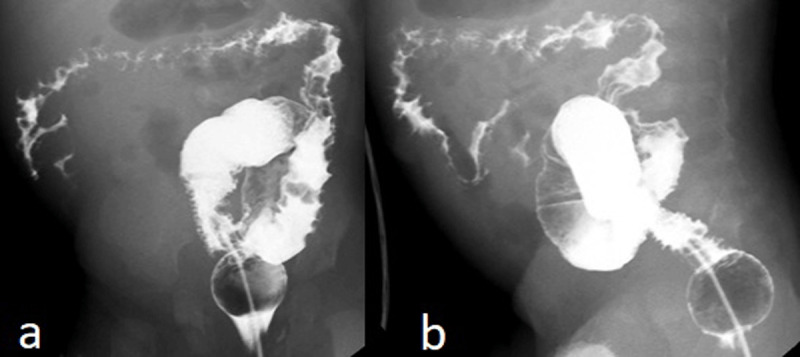
Barium enema – reduced caliber and irregular contour of the lumen of the colon, with *thumbprinting*. The sigmoid colon was relatively spared.

**Figure 2:**
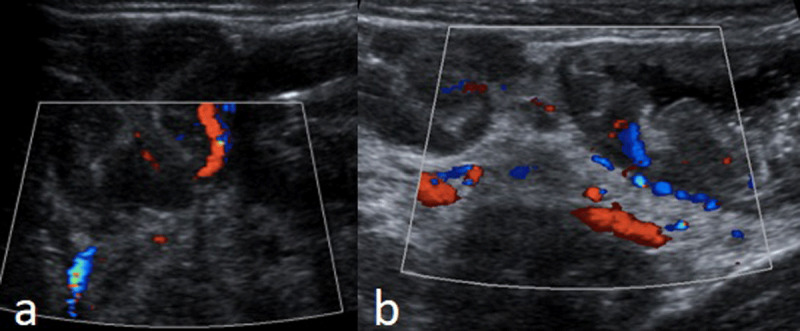
Abdominal ultrasound – diffuse parietal thickening of the colon, with increased vascularization on color Doppler evaluation. The mesentery at the right upper quadrant (b) was also thickened and showed increased vascularization.

**Figure 3:**
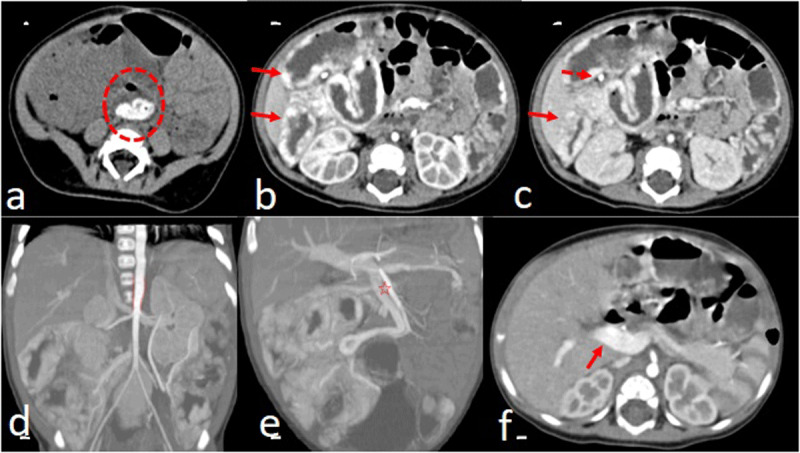
Abdominal CT images – a) axial pre-contrast; b) axial arterial phase; c) axial portal phase; d) coronal MIP arterial phase; e) coronal MIP portal phase; f) axial arterial phase at the level of the portal vein – showed high-density material in the rectum consistent with blood (dashed circle in a) and thickening of the colon wall with intense globular mural enhancement and diffuse filling (arrows in b and c). The mesentery of the right quadrants was also thickened and enhancing, and a small mesenteric phlebolith (dashed arrow in c) was seen. MIP images showed tapering of the aorta at the level of the superior mesenteric artery (faded lines in d) and enlargement of the superior mesenteric artery and vein (star in e). The portal vein was also enlarged and showed avid enhancement in the arterial phase (arrow in f).

Colonoscopy revealed multiple pseudopolipoid lesions with mucosal edema along the colon, sparing the sigmoid and rectum. Barium enema (Figure 1), abdominal ultrasound with color Doppler (Figure 2), and contrast-enhanced computed tomography (CT) with water enema were performed (Figure 3). Histopathological analysis of the colonic biopsies revealed marked capillary ectasias within the lamina propria suggesting multiple hemangiomas.

**Figure 4:**
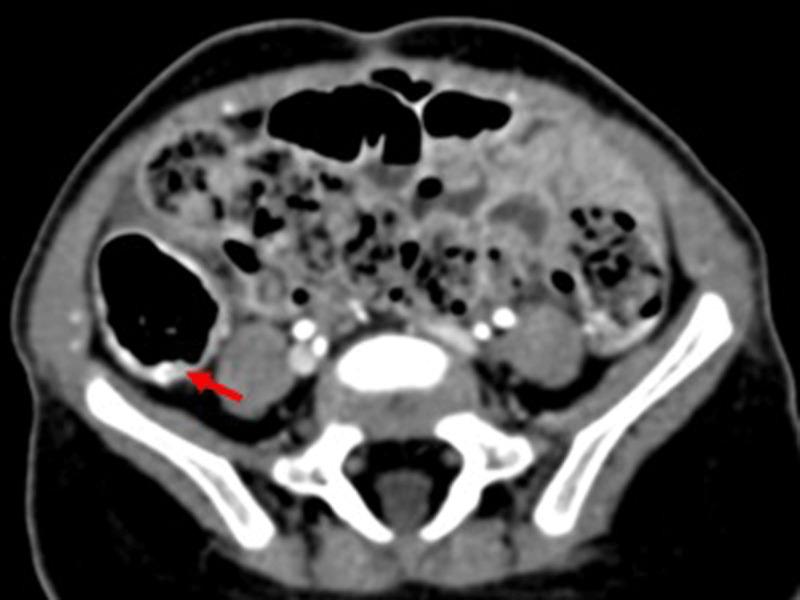
Abdominal CT image of the abdomen (arterial phase) 12 months after treatment with propranolol showed almost complete regression of the bowel wall thickening, with slight arterial phase hyperenhancement on the cecal wall as the only finding.

The infant was diagnosed with gastrointestinal hemangiomatosis and was treated with propranolol for one year. Repeat CT showed regression of the findings (Figure 4), confirmed at endoscopy. During a five-year follow-up no further bleeding was reported.

## Comments

The authors describe this case due to its rarity and successful treatment with pharmacotherapy alone. Infantile hemangiomas are the most common vascular tumours in infancy [1] and are typically cutaneous lesions. Other visceral hemangiomas include the liver, lungs, brain, and intestine [2]. Intestinal hemangiomas are rare, being more common in the small bowel [3,4]. The most common clinical presentation of gastrointestinal hemangiomatosis is gastrointestinal bleeding, which is usually painless and may range from slowly progressive to massive or life threatening [3]. Other manifestations include intussusception, bowel obstruction, anemia or perforation, and congestive heart failure [1,3,5]. Unlike vascular malformations, infantile hemangiomas have the capacity to involute after a proliferative period, requiring no intervention in most of the cases [2]. In addition, multiple hemangiomas may be associated with high-output cardiac failure and coagulopathy [6].

In the past, the diagnosis required exploratory surgery or arteriography [7]. Nowadays, CT and entero-magnetic resonance imaging (MRI) are non-invasive and reliable methods to diagnosis of hemangiomatosis [4]. Positive oral contrast should not be administrated, as it can obscure mural enhancement [8]. CT is also important to evaluate the extra-intestinal findings as hemangiomatosis may infiltrate the mesentery, solid abdominal organs or the retroperitoneum [3]. In addition to colonoscopy, scintigraphy or capsule endoscopy may be useful when the bleeding focus is not detected by other exams.

Propranolol has been described as an effective treatment for infantile hemangiomas, and our patient showed a significant response to propranolol treatment alone [4], which contrasts with previous published series that underwent surgical excision [9,10].
